# A New Human Blood–Retinal Barrier Model Based on Endothelial Cells, Pericytes, and Astrocytes

**DOI:** 10.3390/ijms21051636

**Published:** 2020-02-27

**Authors:** Claudia G. Fresta, Annamaria Fidilio, Giuseppe Caruso, Filippo Caraci, Frank J. Giblin, Gian Marco Leggio, Salvatore Salomone, Filippo Drago, Claudio Bucolo

**Affiliations:** 1 Department of Biomedical and Biotechnological Sciences, School of Medicine, University of Catania, 95125 Catania, Italy; forclaudiafresta@gmail.com (C.G.F.); gianmarco.leggio@unict.it (G.M.L.); salomone@unict.it (S.S.);; 2 Department of Drug Sciences, University of Catania, 95125 Catania, Italy; annafidilio@yahoo.it (A.F.); carafil@hotmail.com (F.C.); 3 Oasi Research Institute—IRCCS, 94018 Troina, Italy; forgiuseppecaruso@gmail.com; 4 Eye Research Institute, Oakland University, Rochester, MI 48309, USA; giblin@oakland.edu; 5 Center for Research in Ocular Pharmacology-CERFO, University of Catania, 95125 Catania, Italy

**Keywords:** diabetic retinopathy, blood–retinal barrier, astrocytes, oxidative stress, inflammation

## Abstract

Blood–retinal barrier (BRB) dysfunction represents one of the most significant changes occurring during diabetic retinopathy. We set up a high-reproducible human-based in vitro BRB model using retinal pericytes, retinal astrocytes, and retinal endothelial cells in order to replicate the human in vivo environment with the same numerical ratio and layer order. Our findings showed that high glucose exposure elicited BRB breakdown, enhanced permeability, and reduced the levels of junction proteins such as ZO-1 and VE-cadherin. Furthermore, an increased expression of pro-inflammatory mediators (IL-1β, IL-6) and oxidative stress-related enzymes (iNOS, Nox2) along with an increased production of reactive oxygen species were observed in our triple co-culture paradigm. Finally, we found an activation of immune response-regulating signaling pathways (Nrf2 and HO-1). In conclusion, the present model mimics the closest human in vivo milieu, providing a valuable tool to study the impact of high glucose in the retina and to develop novel molecules with potential effect on diabetic retinopathy.

## 1. Introduction

The blood–retinal barrier (BRB) is crucial for proper vision, and the loss of the integrity of this physical barrier greatly contributes to the vision loss in retinal diseases such as diabetic retinopathy. The BRB consists of two compartments: the inner BRB, formed by adherents and tight junctions between adjacent retinal capillary endothelial cells; and the outer BRB, composed of retinal pigment epithelial cells, representing a highly selective barrier for molecules and solutes moving from the choroid into the retina [1,2]. With regard to the inner BRB, the retinal endothelial cells are covered by pericytes, which, in turn, are supported by glial cells such as astrocytes. The latter are involved in the maintenance of both retinal neurons and blood vessels and are able to modulate the BRB function through the release of trophic factors and antioxidants in the retinal microenvironment [3]. Astrocytes play also an important role in the maintenance of the BRB since their processes enfold retinal endothelial cells, with a more defined maturation of the tight junction proteins [1,4].

The integrity of BRB is maintained by endothelial tight and adherence junctions, which create a very tight monolayer, so that macromolecules cannot easily penetrate between the cells forming the retinal unit [5]. The loss of tight junctions, connected to the actin cytoskeleton through zonula occludens-1 (ZO-1), leads to the disruption of the inner barrier [6]. The vascular endothelial (VE)-cadherin, a member of the super-family of cadherins, acts as a plasma membrane attachment site for the cytoskeleton and plays a pivotal role in the regulation of endothelial cell behavior [7,8]. The integrity of both classes of the above-mentioned junctional proteins is crucial for the normal settlement of the barrier functionality [9].

Hyperglycemia can contribute to the BRB breakdown that is the hallmark of diabetic retinopathy associated with pericyte death and loss of endothelial junctions [6,10,11]. It is worth noting that, in the last decade, diabetic retinopathy has been shifted from “microvascular disease” towards “neurovascular disease” [12].

High glucose conditions alter several cellular functions affecting, among others, intracellular calcium levels, NADPH oxidase (Nox2) activity, and nuclear factor kappa-light-chain-enhancer of activated B cells (NF-κB) signaling [13,14,15]. Hyperglycemia has also been linked to the over production of inflammatory mediators (e.g., IL-1β, TNF-α, IL-6) and reactive oxygen species (ROS), with a consequent increase in inflammation and oxidative stress [16,17,18,19,20].

Increased levels of ROS and reactive nitrogen species (RNS), including nitric oxide (NO^•^) and superoxide anion (O_2_^−•^), have been observed in the retina of diabetic rats [21,22,23]. Under pathological conditions, inducible nitric oxide synthase (iNOS) and NADPH oxidase (Nox), responsible for NO^•^ and O_2_^−•^production, respectively [24,25], are over-activated in several cell types including endothelial and immune cells [24,26]. The iNOS-mediated NO^•^ production has been associated with the induction of early vascular changes [27], while Nox2-derived ROS are involved in retinal inflammation [28,29,30]. Furthermore, an overproduction of ROS and RNS has been correlated with the BRB breakdown occurring in the early stage of diabetic retinopathy [31].

An important mechanism in the cellular defense against oxidative stress is represented by the activation of nuclear factor (erythroid-derived 2)-like 2 (Nrf2) pathway, which in turn regulates ROS-sensitive genes including heme oxygenase-1 (HO-1) [32,33,34]. With specific regard to astrocytes, the Nrf2 pathway has been shown to play a key role in counteracting oxidative stress-induced cell death [35].

In vitro models of BRB provide a valuable tool to better understand the retinal pathophysiology as well as the trafficking occurring in the barrier [36,37]. In particular, co-cultures of BRB are widely used in order to clarify the cross-talk between the cells of the retinal unit. According to Nakagawa et al., in vitro models of rat blood–brain barrier (BBB), in which endothelial cells and pericytes are co-cultured on opposite sides of a transwell insert and astrocytes are located at the bottom of the culture dish, well represent the in vivo anatomical position of the cells at the BBB [38], which, as is well-known, shares histological and functional similarities with the BRB [39]. Recently, Wisniewska-Kruk et al. [40] set up a BRB model based on a triple co-culture, even though they did not used human cells. These authors employed bovine endothelial cells, bovine pericytes, and rat astrocytes, therefore a bit far from human BRB. In order to set up a better BRB in vitro model closer to the human in vivo environment, we characterized an in vitro human primary culture based on triple co-culture BRB model using human retinal endothelial cells, human retinal pericytes, and human retinal astrocytes, keeping the same cellular layer order present in human, and the same numerical ratio.

In the present study, we first evaluated barrier tightness and paracellular permeability of our BRB system by measuring the trans-epithelial electrical resistance (TEER) and the flux of the water-soluble inert tracer, sodium fluorescein (Na-F). Next, we examined the functional integrity of the BRB by immunofluorescence staining. The effects of high glucose exposure were also assessed by measuring ROS production (fluorescence), and the expression levels of genes related to pro-inflammatory mediators and oxidative stress (quantitative real time PCR (qRT-PCR)). Lastly, we investigated the effect of high glucose exposure on NF-κB and Nrf2, as well as on its downstream gene heme oxygenase 1 (HO-1), by Western blot analysis.

## 2. Results

### 2.1. BRB Integrity

**Figure 1:**
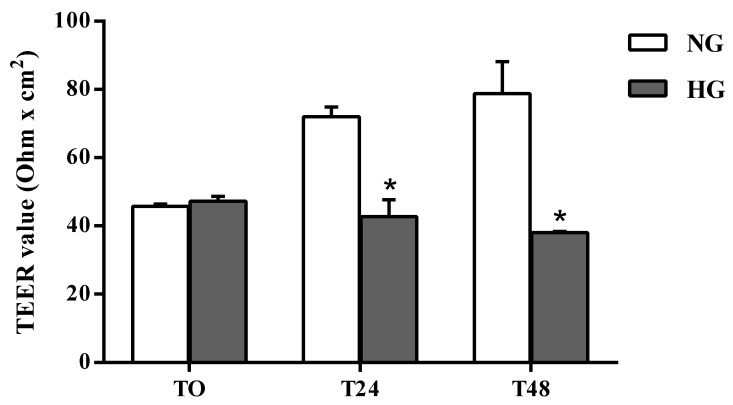
Assessment of barrier integrity in the in vitro human primary culture based triple co-culture BRB model by TEER. TEER values were measured at time 0 (TO), and after 24 (T24) and 48 (T48) h. NG = normal glucose condition (5 mM); HG = high glucose condition (40 mM). Values are means ± standard deviation (SD) of five independent experiments. Two-way ANOVA with Bonferroni’s post-hoc analysis. *p < 0.0001 vs. NG.

The effects of high glucose conditions on BRB integrity were first assessed by performing TEER measurements. As shown in Figure 1 the permeability was significantly altered by high glucose conditions compared to normal glucose (control) conditions at both time points. A reduction of 41% was observed after 24 h (*p* < 0.0001 vs. normal glucose), which further increased (–52%) after 48 h of treatment.

**Figure 2:**
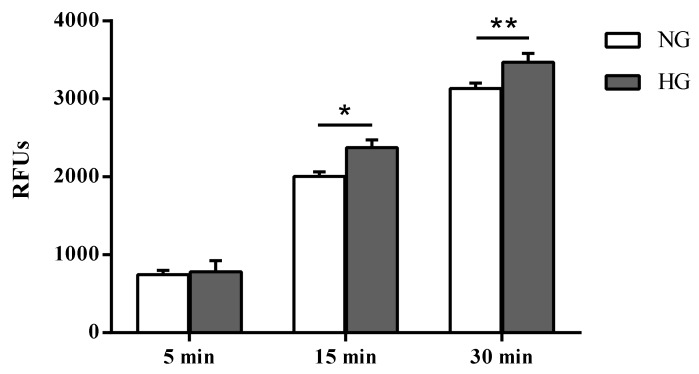
Measurement of the apical-to-basolateral movements of Na-F in the in vitro human primary culture based triple co-culture BRB model. Na-F permeability was measured after 5, 15, and 30 min. NG = normal glucose condition (5 mM); HG = high glucose condition (40 mM). Values, presented as a mean of relative fluorescence units (RFUs), are means ± SD of three independent experiments. Two-way ANOVA with Bonferroni’s post-hoc analysis. *p < 0.01 vs. NG; **p < 0.05 vs. NG.

Paracellular permeability was assessed in cells subjected to normal or high glucose conditions for 48 h using the fluorescent marker Na-F (Figure 2). As expected, an inverse correlation was observed between the TEER values and the Na-F permeability (Figure 2).

Unlike the difference in fluorescence due to Na-F passage (permeability) measured in the two different media collected by cells cultured under normal and high glucose conditions after 5 min (4.9%, not significant), significant differences were observed after 15 and 30 min (*p* < 0.01 and *p* < 0.05 vs. normal glucose, respectively).

### 2.2. ZO-1 and VE-cadherin Levels

**Figure 3:**
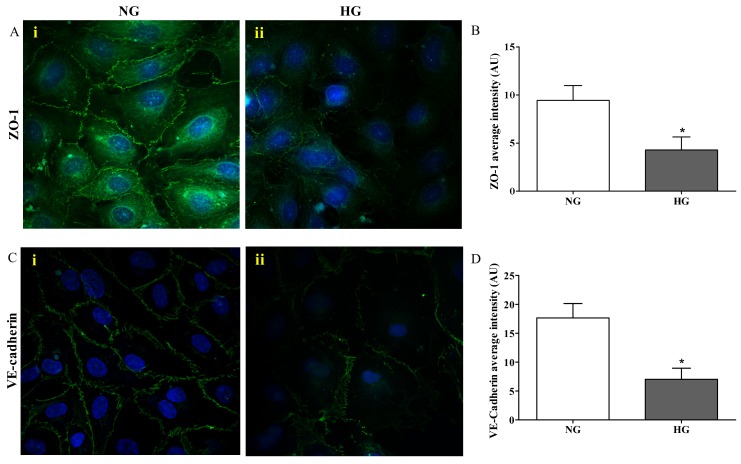
Confocal analysis of ZO-1 (**A**) and VE-cadherin (**C**) in endothelial cells subjected to normal or high glucose conditions for 48 h. ZO-1 and VE-cadherin were labeled with FITC (green) while nuclei were labeled with 4’,6-diamidine-2’-phenylindole dihydrochloride (DAPI) (blue). The continuous brush border showed for normal glucose conditions (A(i) and C(i)) is interrupted under high glucose conditions (A(ii) and C(ii)). The average intensity (AU) of the data from more than 30 cells per coverslip for ZO-1 and VE-cadherin under normal and high glucose conditions are reported in (**B**) and (**D**), respectively. Images for ZO-1 and VE-cadherin immunostaining were acquired at 20 or 60× magnification. NG = normal glucose condition (5 mM); HG = high glucose condition (40 mM). Values are means ± SD of three independent experiments. Statistical analysis was performed using Student’s t-test. *p < 0.001 vs. NG.

Figure 3 depicts the results of the immunocytochemistry analysis performed in the endothelial cells monolayer, part of the in vitro BRB model, grown under normal and high glucose conditions.

The presence of ZO-1 was significantly reduced in cells exposed to high glucose (Figure 3A(ii)) compared to normal glucose conditions (*p* < 0.001; Figure 3A(i)), where distinct ZO-1 staining at the cell–cell borders was observed. The quantification of ZO-1 intensity, measured as fluorescence arbitrary units (AUs), under both normal and high glucose conditions is shown in Figure 3B. It is worthy to note that high glucose exposure affected the presence of VE-cadherin in a similar way to ZO-1. In fact, the staining of VE-cadherin appeared to be markedly reduced and discontinuous in endothelial cell monolayers under high glucose conditions (Figure 3C(ii)), while endothelial cells under normal glucose conditions showed a continuous VE-cadherin brush border (Figure 3C(i)). The quantification of VE-cadherin intensity (AU) under both normal and high glucose conditions, clearly showing the significant difference (*p* < 0.001) between both conditions, is reported in Figure 3D.

### 2.3. Biomarkers of Inflammation and Oxidative Stress

**Figure 4:**
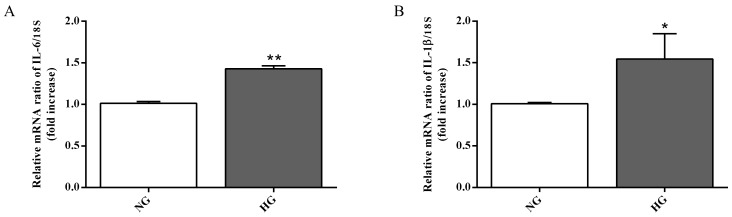
Measurement of (**A**) IL-6 and (**B**) IL-1β mRNA expression levels (qRT-PCR) in astrocytes subjected to normal or high glucose conditions for 48 h. The abundance of each mRNA of interest was expressed relative to the abundance of 18S rRNA, as an internal control. NG = normal glucose condition (5 mM); HG = high glucose condition (40 mM). Values are means ± SD of three independent experiments. Statistical analysis was performed using Student’s t-test. *p < 0.05 vs. NG; **p < 0.01 vs. NG.

Astrocytes are able to modulate the BRB function through the release of trophic factors and antioxidants in the retinal microenvironment [3]. The over-activation as well as the dysfunction of astrocytes represent key contributors to the BRB injury and other retinal vascular diseases [41]. Since high glucose conditions have been shown to compromise retinal astrocytes function [17,42] and to be connected to the inflammatory cytokines secretion and to the induction of neuronal death [43], we measured the expression of two well-known pro-inflammatory cytokines (IL-6 and IL-1β) in astrocytes, part of the in vitro BRB model, grown under normal as well as high glucose conditions. As clearly shown in Figure 4A,B, the expression levels of both pro-inflammatory cytokines were significantly increased by high glucose conditions compared to normal glucose conditions (*p* < 0.01 for IL-6; *p* < 0.05 for IL-1β).

**Figure 5:**
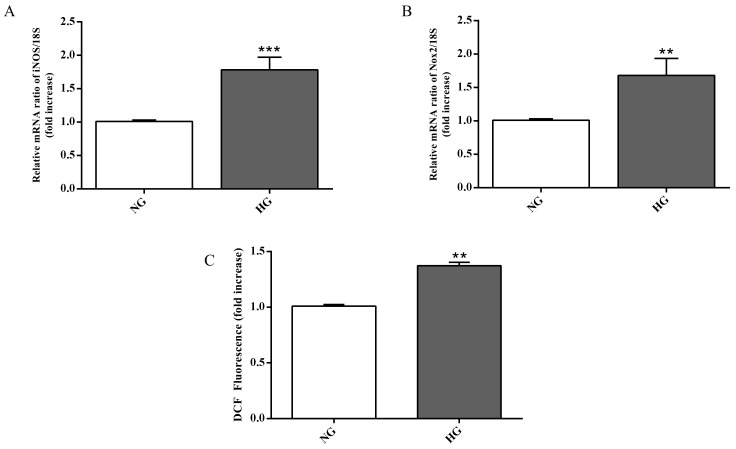
Measurement of (**A**) iNOS and (**B**) Nox2 expression levels (qRT-PCR) in astrocytes subjected to normal or high glucose conditions for 48 h. The abundance of each mRNA of interest was expressed relative to the abundance of 18S rRNA, as an internal control. (**C**) Intracellular ROS production in astrocytes under normal and high glucose conditions. NG = normal glucose condition (5 mM); HG = high glucose condition (40 mM). Values are means ± SD of three to four independent experiments. Statistical analysis was performed using Student’s t-test. **p < 0.01 vs. NG; ***p < 0.001 vs. NG.

In order to assess whether the ability of high glucose conditions to enhance IL-6 and IL-1β expression level is also linked to oxidative stress, we measured the gene expression of enzymes related to the oxidative stress as well as the total ROS production in astrocytes. High glucose conditions significantly raised both iNOS (*p* < 0.001; Figure 5A) and Nox2 (*p* < 0.01; Figure 5B) expression levels compared to normal glucose conditions.

As shown in Figure 5C, high glucose conditions were also responsible for a higher accumulation of intracellular ROS in astrocytes compared to normal glucose conditions (*p* < 0.01).

### 2.4. NF-κB, Nrf2, and HO-1 Levels

**Figure 6:**
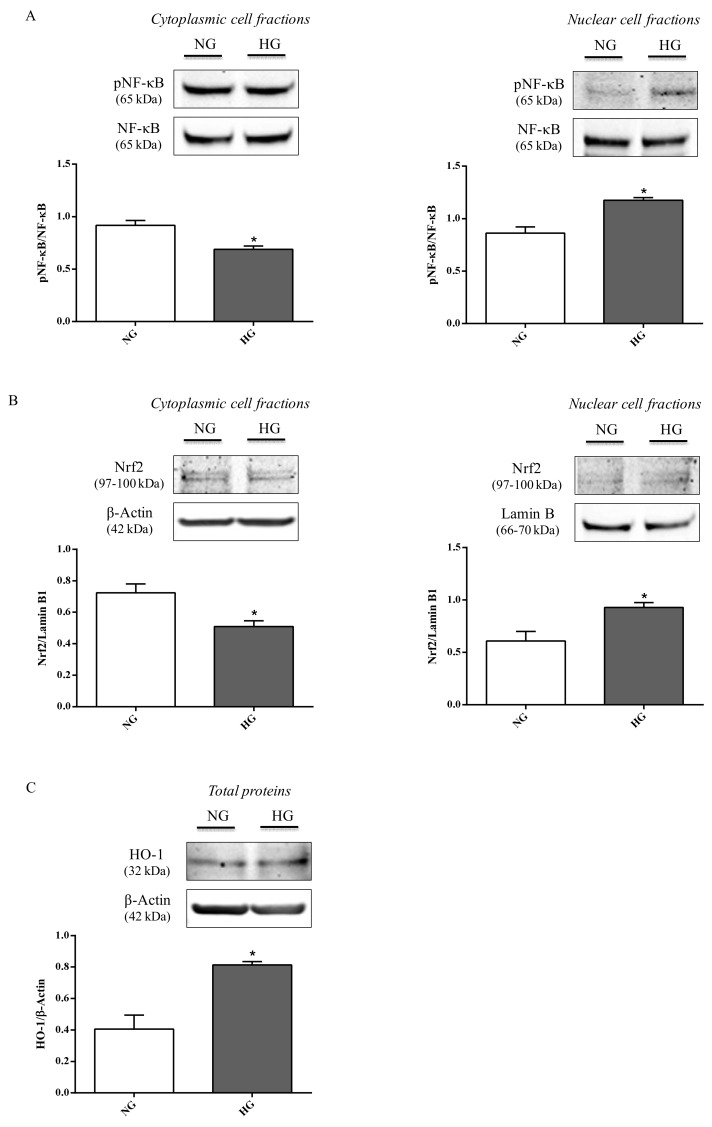
Representative immunoblots of (**A**) cytoplasmic and nuclear pNF-κB, (**B**) cytoplasmic and nuclear Nrf2, and (**C**) total HO-1 in protein extracts from astrocytes subjected to normal or high glucose conditions for 48 h. NG = normal glucose condition (5 mM); HG = high glucose condition (40 mM). Histograms refer to the means ± SD of three independent experiments. Statistical analysis was performed using Student’s t-test. The densitometric values of cytoplasmic and nuclear pNF-κB bands were normalized against total NF-κB. The densitometric values of cytoplasmic Nrf2 and total HO-1 bands were normalized against β-actin, while densitometric values of nuclear Nrf2 bands were normalized against lamin B1. *p < 0.05 vs. NG.

To address whether the propensity to increase inflammation and oxidative stress was also connected to other molecular events, the effect of high glucose conditions on pNF-κB and Nrf2 nuclear translocation as well as HO-1 expression was studied in astrocytes. Compared to normal glucose levels, high glucose conditions promoted an increase in the nuclear protein fraction of pNF-κB (*p* < 0.05; Figure 6A) and Nrf2 (*p* < 0.05; Figure 6B) nuclear translocation, while, as expected, an opposite situation was observed for the cytoplasmic protein fractions (*p* < 0.05 for both of them; Figure 6A,B).

It is also worth underlining that the total levels of both proteins (NF-κB and Nrf2),were not affected by high glucose. Figure 6C shows the significant increment in HO-1 protein levels observed in astrocyte extracts obtained by cells challenged with high glucose conditions compared to normal glucose conditions (*p* < 0.05).

## 3. Discussion

**Figure 7:**
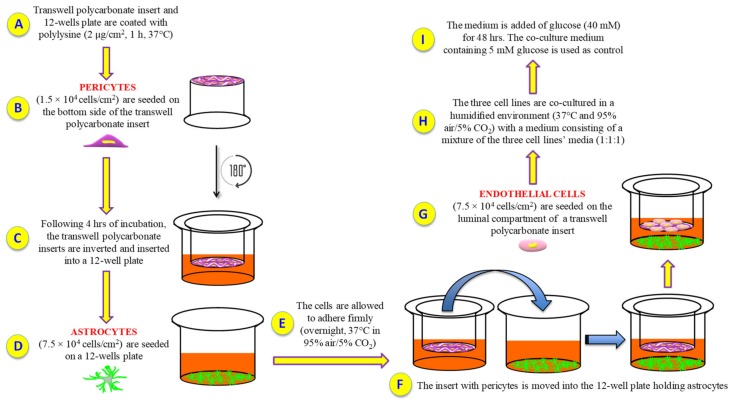
Experimental procedure followed to set up the in vitro BRB model. (**A**) Polylysine (PLL) coating; (**B**) human retinal pericytes are seeded on the bottom side of the insert; (**C**) insert is rotated of 180° and inserted into a 12-well plate containing pericytes medium; (**D**) human retinal astrocytes are seeded on a 12-well plate containing astrocytes medium; (**E**) cell incubation and adhesion; (**F**) the insert with pericytes is moved into the 12-well plate holding astrocytes; (**G**) human retinal endothelial cells are seeded on the top side of the insert; (**H**) cells are grown with a medium consisting of a mixture of the three cell lines’ media (1:1:1); (**I**) glucose was added in order to obtain high glucose conditions.

Cell culture models can be simply set up and represent valuable tools to investigate several biological phenomena occurring on complex systems such as BRB and BBB, under physiological and pathological conditions. Unfortunately, so far there are no BRB triple co-culture models that used human retinal cells. Therefore, the first aim of this study was to characterize a new and reproducible in vitro human primary culture based triple co-culture BRB model based on retinal pericytes, retinal astrocytes, and retinal endothelial cells, mimicking the human milieu [44]. Keeping in mind this goal, we tried to maintain the same position and order of the cellular layers in human. We also investigated the alterations of the inner BRB responsible for the biological retinal modifications occurring in ocular pathological conditions such as diabetic retinopathy. Our BRB co-culture system was build-up by placing endothelial cells and pericytes on the opposite sites of a porous membrane of a trans-well insert, and astrocytes on the luminal compartment of the well (Figure 7).

Once our BRB system was assembled, we evaluated: (i) the overall barrier tightness and paracellular permeability; (ii) the distribution of junctional adhesion molecules in endothelial cells; (iii) the expression of genes related to inflammation and oxidative stress as well as total ROS production in astrocytes; (iv) the protein expression levels of elements of immune response-regulating signaling pathways in astrocytes. In order to better understand the BRB alterations occurring in diabetic retinopathy, we evaluated the above-mentioned parameters in our system under normal (5 mM) and high (40 mM) glucose conditions.

The structural integrity variations of the in vitro BRB model following high glucose insult were assessed after 24 and 48 h, since well-defined BRB damage can be detected at these time points [45]. A significant decrease of TEER values was observed after both a 24 (–41%) and 48 (–52%) h challenge with high glucose exposure as compared with control cells (normal glucose) (Figure 1). The loss of BRB integrity was further confirmed by a significant increase in Na-F permeability from the apical to the basolateral compartment of the insert (Figure 2). The results of the experiments shown in Figure 1 and Figure 2 indicate a leaky retinal barrier under high glucose conditions, in accordance to what has been observed in patients with diabetic retinopathy [46].

Among the tight junction-associated proteins, ZO-1 plays a key role in BRB integrity, and its deficiency has been connected to a setback in the formation of the tight junction protein complex [47,48] and an increased permeability of the BRB in patients affected by diabetic retinopathy [49]. In accordance with the aforementioned, the challenge of the endothelial cells, part of our BRB model, with high glucose exposure caused a robust decrease in terms of ZO-1 protein levels (Figure 3B) as depicted by a discontinuous brush border at the cell–cell contact (Figure 3A(ii)), very different from the distinct ZO-1 staining observed at the cell–cell borders under normal glucose conditions (Figure 3A(i)). We also investigated the VE-cadherin modifications, another protein implicated in the BRB breakdown, during diabetic retinopathy [50,51]. As observed for ZO-1, the immunocytochemistry analysis carried out on endothelial cell monolayers challenged with high glucose showed an evident alteration of the organization pattern of VE-cadherin staining (Figure 3C(ii)) that was significantly different compared to normal glucose conditions (Figure 3C(ii),D). Overall, these results showed that high levels of glucose lead to mechanical disruption of both adherents and tight junction proteins in BRB and then to the loss of cell-to-cell contact in endothelial cell monolayers.

During both early and late stages of diabetic retinopathy, inflammation and oxidative stress take place [52]. It is well known that astrocytes, the major glial cell within the central nervous system, participate in different pathophysiological mechanisms by releasing pro- and anti-inflammatory mediators [53,54]. These cells also play a central role in the development of the retinal vessels and in the maintenance of the BRB [35]. As previously mentioned, high glucose conditions negatively influence the integrity of the BRB, and this phenomenon is due, at least in part, to the ability of high glucose to compromise retinal astrocytes function as well as their morphology and viability [17,42]. Nevertheless, the precise molecular mechanisms leading to astrocyte dysfunction is currently poorly understood and under debate. With the aim to better clarify the role of astrocytes in the context of BRB, we analyzed the expression level of IL-1β, IL-6, iNOS, and Nox2 mRNAs as well as the total ROS production in astrocytes exposed to high glucose levels. Our results showed a significant increase in the expression levels of the above-mentioned pro-inflammatory molecules (IL-1β, IL-6) (Figure 4) and pro-oxidant (iNOS, Nox2) (Figure 5A,B) mediators coupled to the increased production of ROS (Figure 5C). The present data clearly demonstrate that high glucose levels boost the pro-oxidant and pro-inflammatory behavior of astrocytes, part of our BRB system. These results are in agreement with previous findings that showed the link between the inflammatory mediators and the up-regulation of iNOS and Nox2 in in vivo models of diabetic retinopathy [55,56]. Additionally, several papers have shown that hyperglycemia-induced ROS production plays an important role in different pathological conditions such as diabetic retinopathy [57,58,59].

Inflammation and oxidative stress may be related to pro-inflammatory and pro-oxidant mediators increase or to a deficiency and/or impairment of endogenous defense signaling pathways. For this reason the effects of high glucose on NF-κB, Nrf2, and HO-1 protein expression levels in astrocytes were also investigated. By protein extracts of astrocytes, we found that high glucose exposure significantly promoted the nuclear translocation and the following activation of pNF-κB and Nrf2, also enhancing HO-1 expression (Figure 6) compared to normal glucose conditions. This finding is in line with previous data pointing out that: (1) NF-κB represents one of the most common intracellular targets of hyperglycemia [60], and its translocation to the nucleus regulates the expression of several factors including pro-inflammatory cytokines [61]; (2) Nrf2, a transcription factor that is involved in the regulation of ROS-sensitive genes during oxidative stress events, translocates to the nucleus [62] allowing the transcription of genes directly involved in the protection against oxidative stress such as HO-1 [63]. The latter has been shown to exert a protective role in diabetic retinopathy [64].

## 4. Materials and Methods 

### 4.1. Materials and Reagents

Human retinal endothelial cells, human retinal pericytes, human retinal astrocytes, endothelial cell medium (ECM), fetal bovine serum (FBS), endothelial cell growth supplement (ECGS), pericyte cell medium (PM), pericyte supplement factor (PGS), astrocyte cell medium (AM), astrocytes supplement factor (AGS), PLL, and penicillin–streptomycin were purchased from INNOPROT (Derio, Bizkaia, Spain). Cell culture inserts, 75 cm^2^ polystyrene culture flasks, 12-well plates, 96-well plates, and rat-tail collagen type I were obtained from Corning Inc. (Corning, NY, USA). Paraformaldehyde (PFA), normal goat serum (NGS), Triton-X 100, Tween 20, phosphatases and proteases inhibitors, RIPA buffer, D-glucose, and tris buffered saline (TBS) were all supplied by Sigma–Aldrich (St. Louis, MO, USA). Cy3 goat anti-mouse secondary antibody (ab97035), FITC-conjugated goat anti-rabbit (ab97050), anti-von Willebrand factor (ab6994), anti-HO-1 (ab13248) primary antibodies, and 2’,7’-dichlorofluorescin diacetate (DCFDA)—cellular ROS assay kit were purchased from Abcam (Cambridge, UK). Odissey^®^ blocking buffer (PBS) as well as IRDye^®^ 800CW (92632211) and 680LT (92668020) secondary antibodies were obtained from LiCor (Lincoln, USA). Anti-VE-cadherin (2500), anti-phospho-NF-κB p65 (anti-pNFκB) (3033), anti-NFκB (8242), and anti-Nrf2 (12721) primary antibodies were supplied by Cell Signaling (Leiden, Netherlands). Anti-ZO-1 primary antibody (61-7300), mounting medium, TRIzol reagent, SuperScript III first-strand synthesis system for RT-PCR, Pierce™ BCA protein assay kit, Ne-PER nuclear and cytoplasmic extraction reagents, NuPage™ 4%–12% bis–tris gel, and DAPI (D1306) were purchased from Thermo Fisher Scientific (Waltham, MA, USA). Sodium fluorescein (Na-F), anti-β-actin (sc-47778) and anti-lamin B1 (sc-365214) primary antibodies were obtained from Santa Cruz Biotechnology, Inc. (Dallas, TX, USA). Anti-GFAP primary antibody (NB120-10062) was supplied by Novus Biologicals (Milan, Italy). Anti-α-SMA primary antibody (M0851) was purchased from Dako (Santa Clara, CA, USA). 

Nitrocellulose blotting membrane was purchased from GE Healthcare Life Sciences (Amersham, Little Chalfont, UK). LightCycler^®^fast start DNA master SYBR Green I was purchased from Roche Diagnostics (Indianapolis, IN, USA). QuantiTect primer assays were purchased from Qiagen (Hilden, Germany).

### 4.2. Cells

Endothelial cells were grown as previously described [65]. Briefly, the cells were maintained in ECM containing 5% FBS, 1% penicillin–streptomycin, and 1% ECGS under a humidified atmosphere (95% air/5% CO_2_ at 37 °C). Endothelial cells were routinely seeded in a 75 cm^2^ polystyrene culture flask, split at a confluence of 80%–90%, and used for the BRB in vitro model assembly. Cell passage number was always between 2 and 4.

Pericytes were fed with PM supplemented with 100 U/mL penicillin, 100 μg/mL streptomycin, 2% FBS, and 1% PGS. The cells were cultured at 37 °C and in a humidified atmosphere with 95% air/5% CO_2_. As in the case of endothelial cells, pericytes were passaged after reaching 80%–90% of confluence and used for the BRB in vitro model assembly. Cell passage number was always between 2 and 4.

Astrocytes were seeded in PLL-coated 75 cm^2^ polystyrene culture flasks and cultured in AM enriched with 100 U/mL penicillin, 100 μg/mL streptomycin, 2% FBS, and 1% AGS. The cells were maintained in a humidified environment at 37 °C and 95% air/5% CO_2_ and passaged every 3–5 days to avoid cell overgrowth. Astrocytes following the first passage were used for the BRB in vitro model assembly.

### 4.3. BRB Model Set Up

In order to generate the in vitro BRB model, both inserts and 12-well plates were coated with PLL (2 µg/cm^2^) for 1 h at 37 °C followed by three washing steps, two with water and one with PBS. Pericytes were then seeded (1.5 × 10^4^ cells/cm^2^) on the bottom side of polycarbonate inserts and placed upside down. After 4 h of incubation, the inserts were inverted and inserted into 12-well plates containing pericyte medium. During the same day, astrocytes were plated (7.5 × 10^4^ cells/cm^2^) on 12-well plates pre-coated with PLL. The cells were allowed to adhere firmly (overnight, 37 °C in 95% air/5% CO_2_). The following day, the inserts containing pericytes were positioned into the 12-well plates holding astrocytes, while endothelial cells (7.5 × 10^4^ cells/cm^2^) were seeded on the luminal compartment of the inserts having pericytes on the other side. The three cell lines were co-cultured in a humidified environment at 37 °C and 95% air/5% CO_2_ with a medium consisting of a mixture of the three cell lines’ media (1:1:1). Primary cells were co-culture at a ratio of 1:5:5 for pericytes, endothelial cells, and astrocytes, respectively, according to Bonkowski et al. [66], in order to replicate the in vivo numerical ratio. Under the above-mentioned conditions, the in vitro BRB model was established within three days after the setting of the cells. The day of the experiment, the medium was added of glucose (at the final concentration of 40 mM) for 48 h, while the co-culture medium containing a physiological glucose concentration (5 mM) was used as control, as recently described elsewhere [67].

### 4.4. BRB Integrity Assessment

The barrier integrity of the established co-culture was evaluated by TEER measurements using a Millicel-Electrical Resistance System (ERS2) (Merck, Millipore, Burlington, MA, USA) as described elsewhere [68]. Final TEER values of coated filters containing cells, shown as ω × cm^2^, were calculated by subtracting the TEER values of coated cell-free filters. The results, recorded at 0 (T0), 1 (T24), and 2 (T48) days after challenging the cells with high glucose conditions, were multiplied for the surface area (1.12 cm^2^). A 20 min equilibration period at room temperature (RT) was performed prior to the first measurement. TEER values were obtained from five independent experiments.

In order to evaluate the BRB paracellular permeability, we measured the apical-to-basolateral movements of Na-F across endothelial cell monolayers, subjected to normal or high glucose conditions for 48 h, as previously described [38]. After 5, 15, and 30 min, the medium from the lower chamber was collected, and the quantification of fluorescence (Na-F: excitation 480 nm, emission 535 nm) was carried out using a Varioskan Flash microplate reader (Thermo Fisher Scientific, Waltham, MA, USA).

### 4.5. Immunofluorescence Staining

All necessary information of the experimental conditions of the immunofluorescence staining (primary antibodies list, source, dilution, function/characteristics) are reported in Table 1.

Endothelial cells and pericytes were characterized by their positive immunostaining for von Willebrand factor and α-SMA, respectively, as previously described [69] (Figure S1A,B).

In order to characterize the astrocytes, these cells were grown on a PLL-coated coverslip, fixed by using 4% PFA for 20 min at 4 °C, washed three times (5 min each) with 10 mM PBS, and permeabilized with a 10 mM PBS solution containing 5% NGS and 0.1% Triton-X 100 for 30 min at RT. Next, cells were incubated overnight, at 4 °C, with anti-GFAP primary antibody (dilution 1:200). The day after, cells were washed three times with 10 mM PBS and incubated for 1 h, at RT, with Cy3 goat anti-mouse secondary antibody (1:300). As a final step, cell nuclei were stained by using DAPI (1:10,000) (10 min). Cell imaging of GFAP-positive astrocytes (Figure S1C) was performed by using a fluorescence microscope Zeiss Observer Z1 equipped with the Apotome.2 acquisition system connected to a digital camera (Carl Zeiss, Oberkochen, Germany). 

Immunocytochemistry analysis of ZO-1 and VE-cadherin were carried out on endothelial cells under normal and high glucose conditions. In the case of ZO-1, endothelial cell monolayers were washed three times with 10 mM PBS, fixed with ice-cold acetone (100%) at −20 °C for 15 min followed by incubation with ice-cold methanol (100%) at −20 °C for 20 min. Cells were then washed three times with 10 mM PBS and permeabilized with a 10 mM PBS solution containing 5% NGS and 0.1% Triton-X 100 for 10 min at RT. Fixed cells were incubated overnight at 4 °C with ZO-1 antibody (1:100). Washing steps were performed before and after 1 h of incubation with FITC-conjugated goat anti-rabbit antibody (1:300). As a final step, cell nuclei were stained by using DAPI (1:10,000) (10 min).

To assess VE-cadherin, endothelial cells were fixed in 4% PFA and permeabilized with 0.3% Triton-X 100 at RT for 5 min followed by three washing steps. After a blocking step with 1% BSA/10 mM PBS for 1 h at RT, cells were incubated overnight at 4 °C with anti-VE-cadherin antibody (1:100) previously diluted in 10 mM PBS containing 1% BSA. The following day, the coverslips were incubated with FITC-conjugated goat anti-rabbit (1:300) for 1 h followed by PBS washes. 

Semi-quantitative evaluation of ZO-1 and VE-cadherin expression was carried out as previously described [67], with slight modifications. Coverslips were mounted on glass slides by using mounting medium and analyzed with a Leica TCS SP8 confocal laser scanning microscope (Leica Biosystems, Wetzlar, Germany) or Nikon A1RHD25 confocal microscope (Nikon Instruments S.p.A, Florence, Italy). Images for ZO-1 and VE-cadherin immunostaining were acquired at 20× or 60× magnification. The images were analyzed by the use of ImageJ [70] or NIS-Elements software programs. Measurements of average gray scale intensity were carried out at the cell-cell interface in 7 random areas of 7 image rotations. More than 30 cells for each condition were analyzed.

### 4.6. ROS Measurement

Measurement of intracellular ROS in astrocytes, part of the in vitro BRB model, was performed by using a DCFDA cellular ROS assay kit according to the manufacturer’s recommendations. Astrocytes, challenged with high glucose conditions for 48 h, were stained with DCFDA (25 µM, for 45 min at 37 °C), then fluorescence at 485 nm excitation/535 nm emission was measured by employing a Varioskan Flash microplate reader (Thermo Fisher Scientific) to determine total ROS formation. The final fluorescent intensity, after background subtraction, was then normalized to the fluorescent intensity of control cells (normal glucose conditions).

### 4.7. qRT-PCR

Total RNA was extracted from astrocytes, part of the in vitro BRB model, in TRIzol reagent according to the instructions provided by the manufacturer, and re-dissolved in RNase-free water. Reverse transcription of RNA (2 µg) into cDNA was accomplished by using SuperScript III [68]. qRT-PCR was carried out to determine the expression levels of four selected genes by employing LightCycler^®^ FastStart DNA Master SYBR Green I in a final volume of 20 µL (0.5 µM primers, 1.6 mM Mg^2+^, 1X SYBR Green I). 

The QuantiTect Primer Assays (Qiagen) used for gene expression analysis are listed in Table 2, with the exception of IL-1β (forward: 5′-AGC TAC GAA TCT CCG ACC AC-3′; reverse: 5′-CGT TAT CCC ATG TGT CGA AGA A-3′) and 18S rRNA (forward: 5′-AGT CCC TGC CCT TTG TAC ACA-3′; reverse: 5′-GAT CCG AGG GCC TCA CTA AAC-3′) that were purchased by Eurofins MWG Synthesis GmbH (Ebersberg, Germany).

Negative controls (no template control, NTC) were included in each assay. Amplifications were carried out in a Light Cycler 1.5 instrument (Roche Diagnostics, Indianapolis, IN, USA). The relative RNA expression level for each gene of interest was calculated using the 2^–∆∆*C*t^ method [76] by the comparison of the threshold cycle (CT) value of the gene of interest to the CT value of the selected internal control (18S rRNA gene).

### 4.8. Western Blot Analysis

HO-1 protein expression was analyzed by the evaluation of protein concentration in astrocytes, part of the in vitro BRB model, challenged with high glucose conditions for 48 h. Cells were collected and subsequently lysed in RIPA buffer supplemented by phosphatases and proteases inhibitors (1:100). Protein quantification was performed using s Pierce™ BCA protein assay kit, according to the manufacturer’s instructions.

The activation of both Nrf2 and NF-κB on astrocytes under the same experimental conditions described above was measured by employing Ne-PER nuclear and cytoplasmic extraction reagents as previously described in detail [77].

Approximately 25 µg of total, cytoplasmic, and nuclear proteins were separated on 4%–12% tris–glycine gels and transferred to nitrocellulose membranes. The membranes were blotted with anti-HO-1 (1:500), anti-pNFκB (1:1000), anti-NFκB (1:1000), anti-Nrf2 (1:1000), anti-β-actin (1:1000), and lamin B1 (1:1000) primary antibodies in blocking buffer at 4 °C overnight. The day after the membranes were washed three times in TBS/Tween 20, 0.1%, followed by incubation for 1 h with IRDye^®^ 800CW or 680LT secondary antibodies (1:15,000). Bands were visualized using an Odyssey^®^ infrared imaging system (LI-COR Biosciences, Lincoln, NE, USA), and the densitometric analysis was performed by Image J software.

### 4.9. Statistical Analysis

Statistical analysis was performed by using GraphPad Prism 7 (GraphPad Software, La Jolla, CA). Student’s t-test was used to assess the statistical differences between two experimental groups. Two-way analysis of variance (ANOVA), followed by Bonferroni’s post hoc test, was used for multiple comparisons. Only two-tailed *p*-values of less than 0.05 were considered statistically significant. All experiments were repeated at least three times and the data are reported as mean ± SD.

## 5. Conclusions 

We developed the first high-reproducible in vitro BRB model mimicking the inner retinal barrier, entirely based on human cells (retinal pericytes, retinal astrocytes, and retinal endothelial cells). The effects of high glucose exposure were investigated in our BRB model. High glucose elicited remarkable changes in our triple co-culture system; first of all, the high glucose significantly reduced ZO-1 and VE-cadherin in endothelial cells decreasing BRB integrity; secondly, high glucose elicited genes expression related to inflammation and oxidative stress as well as antioxidant response. Taken together, the present findings show that our in vitro paradigm mimics the in vivo human inner BRB, suggesting that this model represents a useful tool for the drug discovery process in ocular pharmacology.

## Figures and Tables

**Table 1:** Details of primary antibodies used for fluorescence immunocytochemistry.

Primary Antibody	Source	Primary Antibody Dilution in PBS + BSA or NGS (1%)	Protein Function/Characteristics
Mouse anti-human α-SMA	Dako ^a^ (M0851)	1:120	Expressed by smooth muscle cells of arterioles and venules, myofibroblasts, and pericytes [71]
Rabbit anti-human von Willebrand Factor	Abcam ^b^ (ab6994)	1:120	Adhesive and multimeric glycoprotein present in blood plasma and produced constitutively in endothelium, megakaryocytes, and subendothelial connective tissue [72]
Mouse anti-human GFAP	Novus Biologicals ^c^ (NB120-10062)	1:200	The hallmark intermediate filament (also known as nanofilament) protein in astrocytes [73]
Rabbit anti-human ZO-1	Life Technology ^d^ (61-7300)	1:100	Scaffold protein located on a cytoplasmic membrane surface of intercellular tight junctions involved in signal transduction at cell–cell junctions [74]
Rabbit anti-human VE-cadherin	Cell Signaling Technology ^e^ (2500)	1:100	Endothelial specific adhesion molecule located at junctions between endothelial cells [75]

Abbreviations: α-SMA, α-smooth muscle actin; GFAP, glial fibrillary acidic protein; ZO-1, zonula occludens-1; VE-cadherin, vascular endothelial-cadherin. ^a^ Dako, Santa Clara, California, USA; ^b^ Abcam, Cambridge, UK; ^c^ Novus Biologicals, Milan, Italy; ^d^ Life Technology, Monza, Italy; ^e^ Cell Signaling Technology, Danvers, MA, USA.

**Table 2:** The list of primers used for quantitative real-time PCR (qRT-PCR).

Official Name ^#^	Official Symbol	Alternative Titles/Symbols	Detected Transcript	Amplicon Length	Cat. No. ^§^
nitric oxide synthase 2, inducible	Nos2	iNOS; Nos-2; Nos2a; i-NOS; NOS-II; MAC-NOS	NM_010927	118 bp	QT00100275
cytochrome b-245, beta polypeptide	Cybb	Cgd; Cyd; Nox2; C88302; gp91-1; gp91phox; CGD91-phox	NM_007807 XM_006527565	146 bp	QT00139797
interleukin 6	Il6	Il-6	NM_031168	128 bp	QT00098875

^#^https://www.ncbi.nlm.nih.gov/gene/; ^§^https://www.qiagen.com/it/shop/pcr/real-time-pcr-enzymes-and-kits/two-step-qrt-pcr/quantitect-primer-assays/.

## Supplementary Materials

The following are available online at https://www.mdpi.com/1422-0067/21/5/1636/s1, Figure S1: The purity of endothelial cells, pericytes, and astrocytes was confirmed by immunofluorescence staining with (A) von Willebrand factor, (B) α-SMA, and (C) GFAP, respectively.
