# Supplementary material for: A New Human Blood–Retinal Barrier Model Based on Endothelial Cells, Pericytes, and Astrocytes
Source: Int J Mol Sci. 2020 Feb 27;21(5):1636. doi: 10.3390/ijms21051636 (PMC7084779; doi:10.3390/ijms21051636)
Supplement: Supplementary file 1 [file ijms-21-01636-s001.pdf]

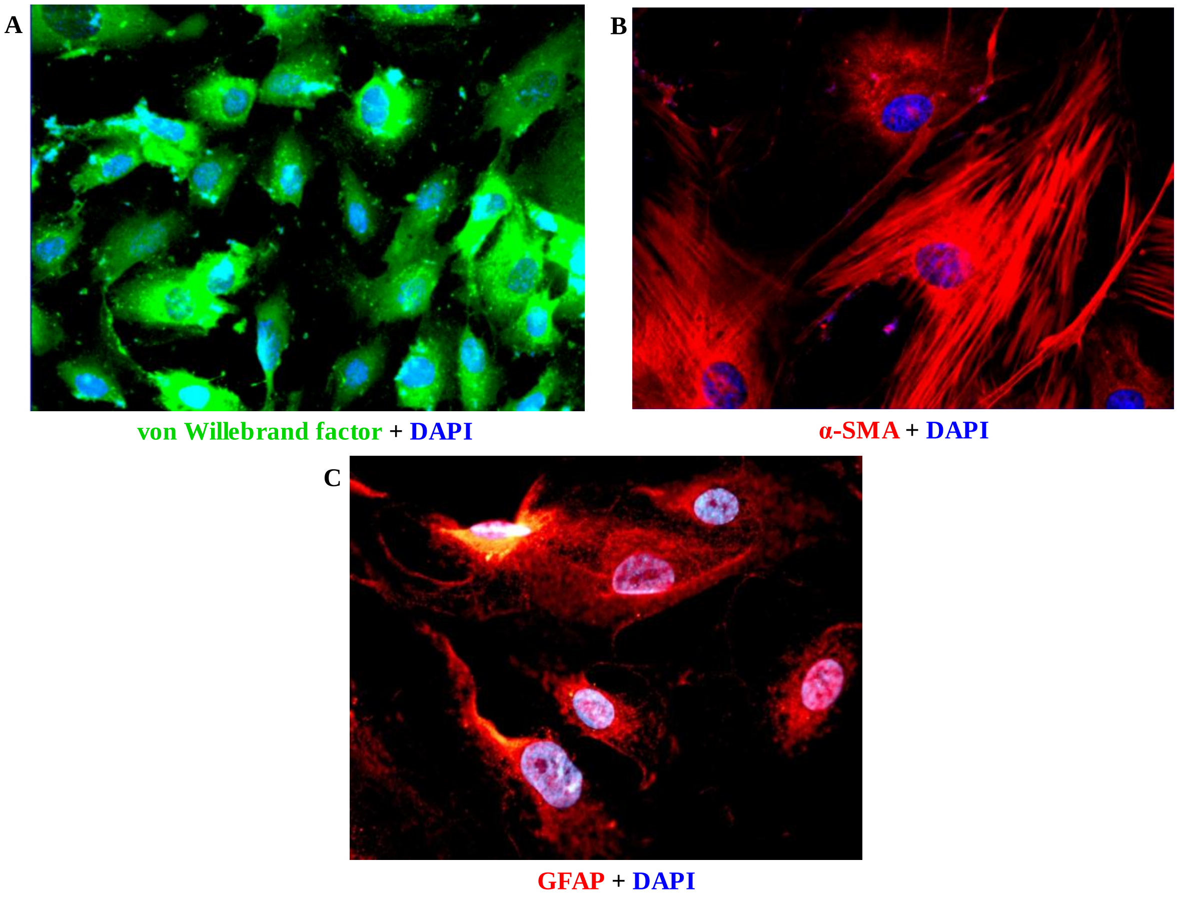

Supplementary Figure 1. The purity of endothelial cells, pericytes, and astrocytes was confirmed by immunofluorescence staining with A) von Willebrand factor, B) *α*-SMA, and C) GFAP, respectively.
